# How to Reprove

**Published:** 1869-05

**Authors:** 


### HOW TO REPROVE.

It requires two accomplished and cultivated persons to give and take reproof, a loving heart and a noble nature, illustrated in the following incident. A nobleman had stopped at a bishop's residence, and won the good will of his host by his courtly address, but he had one bad habit of which the bishop thought he should be informed, "lest it might be to his prejudice." Sending a trusty servant with him when he resumed his journey, he bade him at the right moment to give his friendly warning. It was this, "he had found nothing that was not highly commendable and agreeable except an ugly motion of the mouth and lips when eating, accompanied with a noise very disagreeable to hear." The count, ignorant of his bad habit, blushed, but like a brave man, replied, "Tell the bishop that if all the gifts which men make to one another were like his, men would be much richer than they are. For his great courtesy and liberality to me, I return him infinite thanks, and assure him that I will hereafter guard against my evil habit. God go with you."

